# A simple and robust method for connecting small-molecule drugs using gene-expression signatures

**DOI:** 10.1186/1471-2105-9-258

**Published:** 2008-06-02

**Authors:** Shu-Dong Zhang, Timothy W Gant

**Affiliations:** 1 MRC Toxicology Unit, Hodgkin Building, Lancaster Road, University of Leicester, Leicester, UK

## Abstract

**Background:**

Interaction of a drug or chemical with a biological system can result in a gene-expression profile or signature characteristic of the event. Using a suitably robust algorithm these signatures can potentially be used to connect molecules with similar pharmacological or toxicological properties by gene expression profile. Lamb et al first proposed the Connectivity Map [Lamb et al (2006), Science 313, 1929–1935] to make successful connections among small molecules, genes, and diseases using genomic signatures.

**Results:**

Here we have built on the principles of the Connectivity Map to present a simpler and more robust method for the construction of reference gene-expression profiles and for the connection scoring scheme, which importantly allows the valuation of statistical significance of all the connections observed. We tested the new method with two randomly generated gene signatures and three experimentally derived gene signatures (for HDAC inhibitors, estrogens, and immunosuppressive drugs, respectively). Our testing with this method indicates that it achieves a higher level of specificity and sensitivity and so advances the original method.

**Conclusion:**

The method presented here not only offers more principled statistical procedures for testing connections, but more importantly it provides effective safeguard against false connections at the same time achieving increased sensitivity. With its robust performance, the method has potential use in the drug development pipeline for the early recognition of pharmacological and toxicological properties in chemicals and new drug candidates, and also more broadly in other 'omics sciences.

## Background

One of the most fundamental challenges in all forms of 'omic technologies is the connection of biological event signatures with others previously derived to allow the recognition of new molecule properties or biological alteration. Simple, robust, and efficient matching methods are required to connect a new gene expression signature with those in a database. This problem was first tackled by Lamb et al [1] who introduced the Connectivity Map as a resource and tool to connect small-molecule drugs, genes, and diseases. The Connectivity Map achieved a good degree of success, but also suffered from several deficiencies, particularly an inability to apply a measure of statistical validity at the individual reference signature level to allow rational filtering of the results to exclude false connections. We took the method of Lamb et al as a basis for development and have derived a simple, robust and statistically testable method for making connections between biological event signatures. The method was tested with genomic signatures resulting from small molecule interactions in cells, but also could be applied to any form of signature such as those from proteomic or metabolomic science.

The main assumption behind the concept of a connectivity map is that a biological state, whether physiological, pathological, or induced with chemical or genomic perturbations, can be described in terms of a genomic signature, eg., the genome-wide mRNA levels as measured by DNA microarray technologies. The working of a connectivity map involves several key components. First, a large collection of pre-built reference gene-expression profiles serve as the core database, where each reference profile characterizes a well-defined biological state. Secondly, a query gene signature from some specific studies. A query gene signature is basically a short (as compared to the list of genes in a typical reference profile) list of genes most relevant and important to characterize the biological state of the researchers' interest. Finally, a pattern matching algorithm or similarity metric defined between a query gene signature and a reference gene-expression profile to quantify the closeness or connection between the two biological states. Such a connectivity map can be used by biomedical researchers to find connections between the reference biological states and those of their own interest, leading to testable new biological hypotheses. In this paper, we present a new framework for the construction of reference profiles, new connection scoring scheme and testing procedures for the observed connections. We compare our method with that of Lamb et al, and show that more robust results are achieved using our method. In particular our method not only offers a more principled statistical procedure for testing connections, but more importantly it provides effective safeguards against false connections while at the same time achieving increased sensitivity. As a consequence it can benefit the end users by saving them time and resources in pursuing new biological hypotheses based on the findings of connectivity maps.

## Results

### Construction of reference profiles

For the first-generation connectivity map, Lamb et al carried out a series of gene-expression profiling experiments [1], using 164 distinct small-molecule compounds in a few selected human cell lines. Each treatment instance consisted of one treatment sample and one (or more) vehicle control samples, whose genome wide mRNA levels were measured using Affymetrix GeneChip microarrays. In total 564 samples were microarrayed, which represented 453 different treatment instances. For example, treatment instance ID988 consisted of 1 treatment sample and 6 vehicle control samples. The treatment sample was obtained by treating human MCF7 cells with 100 *nM *estradiol for 6 hours. The control samples were obtained by treating MCF7 cells with vehicle control for 6 hours. A gene-expression profile was constructed for each treatment instance, in which the relative expression (treatment relative to the control) of all the measured genes were specified, and sorted in descending order. A query gene signature, obtained and ordered in the same manner, can be compared to each reference profile in the Connectivity Map to calculate a connectivity score. For "*positive connectivity*", the up-regulated genes of the query signature find matches near the top of the reference profile, and the down-regulated genes find matches near the bottom of the reference profile. For "*negative connectivity*", the matches are opposite.

We obtained Lamb et al's data set (Accession Number GSE5258) from the GEO (Gene Expression Omnibus) database, and rebuilt the 453 reference gene-expression profiles using a new ranking scheme based on the following guiding principles: (1) A treatment instance was defined relative to a control, thus the effect of the treatment could be characterized by the relative differential expression status of all the genes together, (2) different genes were affected to different extents by the treatment, so genes which showed a greater differential expression should have more weight in characterizing this treatment, and (3) up- and down-regulated genes should be treated equally in a unified manner. This meant that a 2-fold down-regulated gene was considered as equally important as a 2-fold up-regulated gene in defining a reference profile. There are several reasons for the choice of treating up- and down-regulated genes equally. Theoretically, unless we have a lot of further information about so many genes on the microarray it is difficult to decide whether this 2-fold up-regulated gene is more important than that 2-fold down-regulated gene or the opposite. So it is logical to assign them equal weights. Another reason is the consideration of symmetry: if a gene is 2-fold up-regulated in sample 1 versus sample 2, it can also be viewed as 2-fold down-regulated in sample 2 versus sample 1. We should emphasize that assigning two genes equal weights does not imply in any sense they share the same molecular mechanism. Even two up-regulated genes with the same fold change could be involved in very different molecular mechanisms. To adhere to the above guidelines, an obvious choice for organizing the genes is the logarithm of the expression ratio (treatment over control). Thus instead of treating the down- and up-regulated genes separately as in the method of Lamb et al, we ordered genes in a reference profile by the absolute value of their expression log-ratios. Therefore the most differentially expressed genes (either up or down) appear first in the list, and those non-differentially expressed genes appear at the bottom of the list. In this way, the genes are ordered by their importance in defining the reference profile. It is then straightforward to assign ranks to them. Suppose there are in total *N *genes, the first gene in the list will be assigned a rank *N *if it is up-regulated, or a rank *-N *if it is down-regulated. In general the *i*th gene in the list will be ranked with (*N - i *+ 1) for up-regulation or *- *(*N - i *+ 1) for down-regulation. With this new ranking method, the importance of a gene is reflected by the absolute value of its rank, while the sign of its rank indicates its regulation status. The consequence and advantage of this method for creating reference profiles is that attaching statistical significance to the connection observed is a relatively straightforward step.

### The scoring scheme

A query gene signature can be an ordered gene list, or just a collection of genes without specific ordering. We will refer to these two types of query gene signatures as *ordered *and *unordered *gene signatures respectively. For an ordered gene signature, we rank the genes in the list in the same way as for a reference profile. Namely, the most important (differentially expressed) gene in the signature will be assigned a rank *m *or *-m *depending on its regulation status, where *m *is the number of genes in the signature. While the least important gene in the signature be ranked 1 or -1.

Let **R **denote a reference gene-expression profile, and **s **a query gene signature. We define the *connection strength *between **R **and **s **as

(1) $$C(R,s)=\sum_{i=1}^{m}R(g_{i})s(g_{i}),$$

where *g*_*i *_represents the *i*th gene in the signature, *s*(*g*_*i*_) is its signed rank in the signature, and *R*(*g*_*i*_) is this gene's signed rank in the reference profile. It is worth noting some properties of the connection strength defined above: (1) if a gene has the same regulation status (either up- or down-regulation) in both the reference and the query, it will make a positive contribution to the connection strength, otherwise its contribution will be negative; (2) the magnitude of a gene's contribution to the connection strength is determined by its position in both lists; and (3) a gene signature with some of its genes contributing positively and others negatively will have an overall low connection strength, because the positive and negative contributions cancel each other to some extent. Therefore when calculating the connection strength between a gene signature and a reference profile, the maximum connection strength achievable is the situation where the *m *genes in the signature match the first *m *genes in the reference profile in the correct order, and their regulation status also match. In such a case, the maximum positive connection strength is, for an *ordered *gene signature,

(2) $$C_{max}^{o}(N,m)=\sum_{i=1}^{m}(N-i+1)(m-i+1).$$

In another equally interesting situation, where the *m *genes in the signature match the first *m *genes in the reference profile in the right order, but the sign of each gene in the query is different from its sign in the reference, the connection strength is $-C_{max}^{o}(N,m)$, the opposite of Eq.(2).

For an unordered query signature, all the genes in the list have equal weight because there is no particular ordering among them. The calculation of connection strength is the same as Eq.(1), the only difference being that *s*(*g*_*i*_) = 1 if gene *g*_*i *_is up-regulated, or *s*(*g*_*i*_) = -1 if it is down-regulated. Consequently, the maximum magnitude of connection strength for an *unordered *signature is

(3) $$C_{max}^{u}(N,m)=\sum_{i=1}^{m}\left(N-i+1\right).$$

Given a query signature gene and a reference gene-expression profile, we can calculate their connection score by

(4) *c *= *C*(**R**, **s**)/*C*_*max*_(*N*, *m*).

So a connection score *c *= 1 means that the gene signature has the maximum positive connection strength with the reference profile, which indicates that the experimental condition that gave rise to this gene signature had the strongest possible correlation with the treatment instance that generated the reference profile. A connection score *c *= -1 indicates that the two experimental perturbations were most inversely correlated. In general, a connection score *c *will be within the range of (-1, 1).

### Connection Testing

As for most biomedical experiments with unavoidable biological and technical variation, statistical significance is a crucial aid to the interpretation and subsequent validation of the result. Here we propose calculating the p-value associated with a connection score by testing the following null hypothesis.

**Null hypothesis ***H*_0_: For a reference gene-expression profile **R **and a query gene signature **s** of length *m*, the null hypothesis *H*_0 _states that there is no underlying biological connection between the two, and that the query signature **s **is merely a random *m*-gene signature, as generated by Procedure 1 described below.

**Procedure 1: **Generation of a random *m*-gene signature. Let **R **be a given reference gene-expression profile of *N *genes. Select *m *genes sequentially and randomly from the *N *genes (without replacement), and assign +1 (up-regulation) or -1 (down-regulation) randomly with equal probability to each of the *m *selected genes. If this gene signature is to be used as an ordered list, its order is just the order in which the *m *genes are selected; or if this gene signature is to be used as merely a collection of genes, then the order is irrelevant.

Given a reference profile **R **and a gene signature **s**, we calculate their connection score *c *by Eq.(4), and the two-tailed p-value associated with this observed connection score is

(5) $$p=\text{Prob }\{\left|\tilde{c}\right|\geq \left|c\right||H_{0}\},$$

where $\tilde{c}$ is the connection score between a random gene signature and the reference profile. To estimate the p-value, a large number (eg., 10^5^) of random gene signatures of the same length *m *can be generated using Procedure 1 and their connection scores to reference profile **R **calculated using Eq.(4), the proportion of random scores that are no less than the observed scores *c *in absolute value is an estimate of the two-tailed p-value.

The 453 individual treatment instances of the data set GSE5258 were created using only 164 distinct small-molecule compounds. Some treatment instances were replication experiments using the same compound at the same or different doses. It is thus interesting to consider all the treatment instances of the same compound as a set, and to assess the overall connection of the set with a query gene signature.

We define the connection score for a treatment set as follows

(6) $$t=\frac{1}{n}\sum_{i=1}^{n}c_{i},$$

where *n *is the number of individual treatment instances belonging to the treatment set, *c*_*i *_is the connection score of the *i*th instance. To test the significance of a treatment set as a whole. We used the following null hypothesis,

**Null hypothesis **$H_{0}^{set}$: Where *T *denotes a set of treatment instances, **R**_**i **_the reference profile based on treatment instance *i*, and **s **a query gene signature of length *m*, the null hypothesis $H_{0}^{set}$ states that there is no underlying biological connection between the gene signature **s **and any of the reference profiles in T. The query signature is merely a random *m*-gene list generated by Procedure 1.

Thus the null hypothesis $H_{0}^{set}$ is an extension of *H*_0 _to a higher level. Alternatively, *H*_0 _can be viewed as a special case of $H_{0}^{set}$, in which there is only one treatment instance in the treatment set. Once the connection score for a set is observed, its associated p value can be estimated in a similar way: a large number of random gene signatures of the same length *m *are generated using Procedure 1, and the connection score of the set to each of the random gene signatures is calculated using Eq.(6); the proportion of random connection scores that are greater than the observed score in absolute value is an estimation of the p value.

### Testing with random gene signatures

To compare the specificities of the original Connectivity Map and the method presented here, we generated random gene signatures and tested these random gene signatures in both. The first example was a random gene signature, rds01, which contained 25 Affymetrix probe-set IDs randomly selected from the 22283 IDs on the Affymetrix HG-U133A microarray platform. Querying the Connectivity Map with this signature, we obtained the connectivity scores of rds01 to all the 453 individual treatment instances. The results are shown in Table 1, where 113 individual reference profiles have positive connectivity scores ranging from 1 to 0.342; 83 individual reference profiles have negative connectivity scores ranging from -1 to -0.38; and the remaining 257 reference profiles have a null connectivity score 0. However, there is no p value or other statistical significance measure associated with these connectivity scores, so users cannot effectively control possible false connections. In this case, because rds01 was a random gene signature, all these positive and negative connections must be false, including the top ones with connectivity scores +1 and -1. So, regardless of what cut-off score is used to call significant connections, all such declared significant connections are false.

**Table 1:** Results for rds01 using the Connectivity Map.

rank	ID	compound	score	up	down
1	1080	sirolimus	1	0.232	-0.578
2	913	colforsin	0.953	0.245	-0.527
3	1138	phentolamine	0.912	0.316	-0.423
4	1048	alpha-estradiol	0.886	0.324	-0.394
5	1115	phenanthridinone	0.869	0.379	-0.325
...	...	...	...	...	...
112	885	5186223	0.363	0.137	-0.157
113	3	metformin	0.342	0.158	-0.119
114	663	U0125	0	0.405	0.194
115	124	mesalazine	0	0.371	0.256
...	...	...	...	...	...
369	1008	geldanamycin	0	-0.43	-0.339
370	1064	17AAG	0	-0.436	-0.395
371	605	monastrol	-0.38	-0.114	0.177
372	494	fluphenazine	-0.392	-0.113	0.187
...	...	...	...	...	...
449	601	MK-886	-0.834	-0.303	0.336
450	604	arachidonic acid	-0.855	-0.301	0.354
451	387	estradiol	-0.901	-0.162	0.528
452	379	cobalt chloride	-0.916	-0.238	0.464
453	378	tacrolimus	-1	-0.23	0.536

The connectivity scores of the random gene signature rds01 to the reference profiles in the Lamb Connectivity Map. The full tabulated results can be found in the supplementary data [see Additional file 1]. 113 reference profiles have positive connectivity scores, 83 reference profiles have negative connectivity scores.

We then used this same random signature to test the new method presented in this paper (Table 2). With a p value calculated for each observed connection score, we can control the expected number of false connections by setting an appropriate threshold p value. In this paper, the threshold p value is generally set at 1/*N*, where *N *is the number of null hypotheses being tested simultaneously. In this example, *N *= 453 (the total number of individual treatment instances in the database and hence the number of null hypothesis being tested at the treatment instance level). So a connection with a p value *p *< 1/453 = 0.0022 is considered as statistically significant. The setting of the threshold p value at 1/*N *was intended to control the expected number of false connections at 1, we thus expected to have one false connection on average among all the connections declared as significant. Table 2 shows that our method gave the correct result, ie., no significant connection between this random signature and any of the treatment instances. Note that to control the expected number of false connections more precisely, the threshold hold p value should be set at 1/*N*_0_, where *N*_0 _is the number of true null hypothesis. Of course in a general situation *N*_0 _is unknown, so it has to be estimated, for example, using the methods developed in [2,3]. In this paper, we set the threshold p value at 1/*N *for simplicity. Since *N*_0 _≤ *N*, our criteria tend to be slightly conservative, meaning that the actual number of false connections on average will be ≤ 1. The second random gene signature, rds02, consisted of 189 Affymetrix probe-set IDs randomly selected from 22283. The full results of querying the Connectivity Map with this signature can be found in the supplementary data [see Additional file 1], where 107 reference profiles have positive connectivity scores ranging from +1 to +0.384, 119 reference profiles have negative connectivity scores ranging from -1 to -0.383, and the remaining 227 reference profiles have a null connectivity score 0. However, once again we know these positive and negative connections must be all false, including the top ones with connectivity scores +1 and -1. In contrast, the method presented in this paper gave results that agree with the known truth. With the criteria set above there was 1 connection found significant (*p *< 1/453 = 0.0022). Since we expect on average 1 false connection, this declared significant connection can be taken as false.

**Table 2:** Results for rds01 using the new method.

Rank	Compound	ID	score	pvalue
1	(-)-catechin	1101	0.334	0.003
2	sirolimus	1022	0.312	0.006
3	phentolamine	1138	0.309	0.007
4	5162773	892	0.292	0.011
5	resveratrol	595	0.286	0.013
...	...	...	...	
451	felodipine	848	-0.002	0.988
452	estradiol	988	-0.001	0.991
453	bucladesine	959	0.001	0.993

The connection scores for the random gene signature rds01 using the new method. To control the expected number of false connections at 1, the threshold p value was set at 1/453 = 0.0022. No connection was found to be significant, which agrees with the known truth.

The two examples of random gene signatures above showed that on an individual treatment instance level, the Connectivity Map does not provide effective safeguards against possible false connections. On the treatment set level, the Connectivity Map provides a permutation p value when a set of treatment instances associated with the same compound were viewed as a whole. The significance of the set of treatment instances in the ordered list of all instances was estimated by permutation (see the Supporting Online Material for [1]). Note that the null hypothesis $H_{0}^{set}$ we use in our set-level analysis is different from that of the Connectivity Map. Tian et al were among the first groups of authors who made explicit distinctions between two different null hypotheses [4] concerning a set of entities (a set of genes in context of Tian et al's paper). Other authors also addressed this issue in some recent studies on the significance analysis of gene sets [5,6]. A more detailed comparison between the null hypotheses used by the set-level analysis of the Connectivity Map and the method presented here can be found in the supplementary information [see Additional file 2].

Throughout this paper, when we use the permutation p values from the Connectivity Map to control false connections, the same criteria discussed above for setting threshold p value are used. The full tabulated results of significance analysis on the connections between the two random gene signatures and the 164 treatments sets can be found in the supplementary data. Both our method and the Connectivity Map gave the right answers for these two random gene signatures, ie, no significant connections were found more than expected by chance. Therefore the set-level analysis of the Connectivity Map can provide a control over possible false connections by means of permutation p values. So in the subsequent analysis on the experimentally derived gene signatures, we only use the set-level results of the Connectivity Map, but not its instance-level results. We need to point out that, in the Connectivity Map set-level analysis, permutation p values were not available for all the 164 treatment sets. For those treatment sets which only contain 1 treatment instance or those sets with mean connectivity score 0, no permutation p values could be calculated, and hence no statistical significance is attached to them. This problem affects the coverage and consequently the sensitivity of the Connectivity Map, because real biological connections between a query gene signature and any of those treatment sets may not be recognized.

### Testing with experimentally derived gene signatures

#### HDAC inhibitors

To test the ability of the new method for identifying real biological connections we utilized some of the same examples used in [1] to compare with the Connectivity Map. The first example was a gene signature of histone deacetylase (HDAC) inhibitors (Lamb's gene signature sigs01), which was compiled from an independent study [7] on the responses of T24, MDA435 and MDA468 cells respectively to three histone deacetylase (HDAC) inhibitors: vorinostat, MS-27–275, and trichostatin A. This gene signature consisted of 8 up- and 5 down-regulated genes, represented by 25 Affymetrix probe-set IDs on the Affymetrix HG-U133A microarrays.

As the Connectivity Map does not provide effective safeguards on individual treatment instance level against possible false connections, we can only use the results of the Connectivity Map on the treatment set level. In total 6 compounds (vorinostat, trichostatin A, resveratrol, geldanamycin, valproic acid, and 17AAG) were identified to have significant positive connectivity with the HDAC inhibitor gene signature; and 2 compounds (5182598 and fludrocortisone) had significant negative connectivity. Vorinostat, trichostatin A, and valproic acid are known HDAC inhibitors thus the identification of these can be regarded as a success of the Connectivity Map. However another known HDAC inhibitor HC-toxin, a reference profile of which was contained in the database, was not identified. This happened because there was only one treatment instance of the compound HC-toxin in the database and so no permutation p value could be obtained using the Connectivity Map. Based on their instance level results, Lamb et al highlighted HC-Toxin in [1] as it had the 7th highest connectivity score (0.914) of all instances in the dataset. However, the two examples of random gene signatures already showed that at the instance level the Connectivity Map gave false connections even for the highest connectivity scores +1 and -1. So the rational choice is to disregard the instance level results from the Connectivity Map.

**Figure 1:**
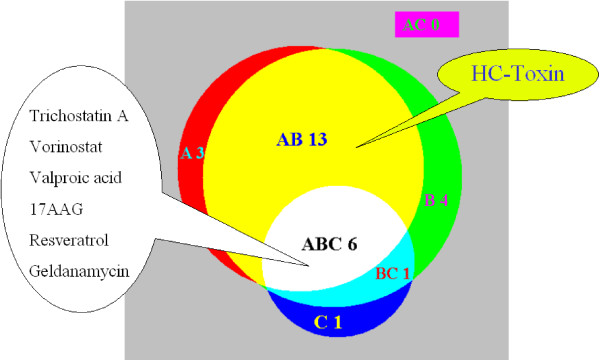
**Results for the HDAC inhibitor gene signature**. The Venn diagram summarizing the findings of significant connections. A represents the instance-level analysis using our new method; B, the set-level analysis using the new method; C, the set-level analysis using the Connectivity Map. The label "AB 13" means that 13 compounds are identified as significant solely by A and B (not C), "B 4" means that 4 compounds are identified as significant solely by B (not A, not C), and so on. The areas are approximately proportional to the numbers they represent.

We used this same HDAC inhibitor gene signature to test the new method presented here, with p values calculated at both the individual instance level and the treatment set level. On the individual instance level, 56 treatment instances, representing 22 distinct compounds, were found to have significant connections to sigs01. On the treatment set level, 24 compounds were found to have significant connections with the signature. Near the top of the significant connection list were those known HDAC inhibitors highlighted in [1]. Importantly though also included in the output was HC-Toxin, which was not identified by the set-level analysis of the Connectivity Map. The full tabulated results for the HDAC inhibitor gene signature are included in the supplementary data. In Fig. 1, we summarize the number of significant connections as identified by: (A) Instance level analysis using the new method presented here; (B) Set level analysis using the new method; (C) Set level analysis using the Connectivity Map. In total, our method (A) and (B) combined identified 27 compounds, while the Connectivity Map identified 8 compounds, as having significant connections to the HDAC inhibitors. Our method missed only 1 of the 8 compounds found significant by the Connectivity Map, while the latter missed 20 of the 27 compounds identified by our method, with HC-toxin among the 20 compounds that were missed. The HDAC inhibitors example thus shows that our method has a greater sensitivity for detecting real connections. With the increased sensitivity and false connections being properly controlled, the potential benefit of our method is obvious. In this example, the set-level analysis of the Connectivity Map identified 8 compounds with a false discovery rate of 12.5% (1/8), while the set-level analysis using our method identified 24 compounds with a false discovery rate of 4.2% (1/24). Based on the findings of significant connections, researchers can prioritize a small sub set of those compounds for further investigations and/or developing new biological hypotheses. For this example, using the Connectivity Map, the chance of pursuing a false connection is 12.5%, while using our method it is much lower at 4.2%. In practice this would save time and resources and increase the rate of success.

#### Estrogens

The second example was a gene signature (Lamb's gene signature sigs02) taken from an independent study [8] of MCF7 cells treated with estradiol. This gene signature consisted of 40 up- and 89 down-regulated genes represented by 189 Affymetrix probe-set IDs on the Affymetrix HG-U133A microarrays. We tested the Connectivity Map and the method presented in this paper with this estrogen signature.

For the same reason given above, we only used the treatment set level results of the Connectivity Map, which identified 4 compounds (genistein, estradiol, tretinoin, and alpha-estradiol) as having significant positive connectivity with the estrogen signature; and 5 compounds (trichostatin A, fulvestrant, LY-294002, vorinostat, and geldanamycin) that had significant negative connectivity.

Using our set-level analysis 16 compounds were found to have significant positive connection, and 25 compounds had significant negative connection to the estrogen gene signature. The 16 compounds with positive connection included genistein, estradiol, and alpha-estradiol, all known to be estrogen receptor agonists. The 25 compounds with negative connection included fulvestrant, raloxifene and tamoxifen, all known to be estrogen receptor antagonists. In comparison, the Connectivity Map identified all the estrogen receptor agonists above, but missed all the estrogen receptor antagonists except fulvestrant. These results therefore indicate the sensitivity of our method is substantially increased. The Connectivity Map was able to detect the pure estrogen receptor antagonist fulvestrant, but missed the two compounds tamoxifen and raloxifene which have mixed antagonist and agonist estrogen receptor activities.

**Figure 2:**
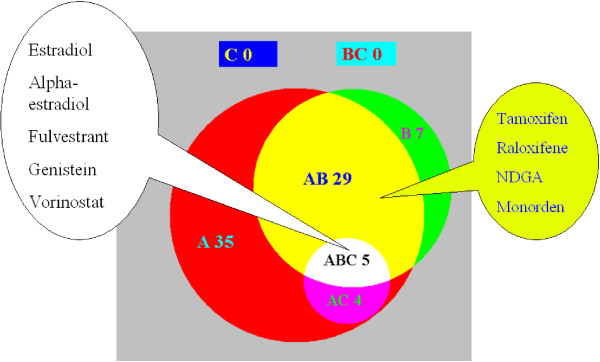
**Results for the Estrogen gene signature**. The Venn diagram summarizing the findings of significant connections as identified by the Connectivity Map and the new method here. The labelling follows the same conventions as in the previous figure.

The results from our set level analysis also suggest that nordihydroguaiaretic acid (NDGA) has significant positive connection with estradiol. This connection is supported by recent studies [9,10], where NDGA has been shown to have estrogenic activity and able to elicit an estrogen-like response. Another compound monorden (radicicol), suggested by our method as having negative connection to the estrogen gene signature, has been shown to repress the transcriptional function of the estrogen receptor [11] which suggests that it may have some estrogen receptor antagonist-like properties. The full tabulated results for the estrogen gene signature are included in the supplementary data. Fig. 2 summarizes the numbers of significant connections identified by our method and by the Connectivity Map. All 9 compounds found significant by the Connectivity Map were also identified by the our method (either on the instance level or on the set level or both). However many compounds identified as significant with either positive or negative connection to estradiol using our method were not identified by the Connectivity Map, included amongst these were raloxifene, tamoxifen, monorden, and NDGA.

#### Immunosuppressive therapy

For further testing we compiled a new gene signature from an independent study on cardiac allograft rejection and response to immunosuppressive therapy [12], where patients were treated with standard immunosuppression with corticosteroids, antimetabolites, calcineurin inhibitors, and/or sirolimus. This gene signature consisted of 40 Affymetrix probe set IDs (see Table 2 of [12]). Our set-level analysis identified 29 compounds as having significant connections with this gene signature. The three top compounds were azathioprine, thalidomide, and rosiglitazone. Azathioprine is a commonly used immunosuppressive drug [13,14], so its significant positive connection with the gene signature is a good indication that the new method works very well here. The second compound thalidomide, which had a positive connection score, also has known immunosuppressive activities [15], inhibits release of TNF*α *from monocytes, and modulates other cytokine actions. The recognized properties of these molecules therefore accord with the outcome of the connectivity matching. The third compound rosiglitazone had a negative connection with the signature suggesting it may have properties to reduce or mitigate the effects of immunosuppressive activity. Recently, rosiglitazone was reported to suppress cyclosporin-induced chronic transplant dysfunction and prolong survival of rat cardiac allografts [16], where cyclosporin is also a commonly used immunosuppressive drug [17].

At the instance level, our method identified 89 reference profiles as having significant connections to the immunosuppressive drug gene signature, representing 63 distinct compounds. The top 3 compounds were azathioprine, staurosporine, and trichostatin A, which all achieved positive connection scores with this gene signature. The second compound, staurosporine, a protein kinase C inhibitor, is classified as an antineoplastic and immunosuppressive antibiotic drug [18]. The third compound, trichostatin A, was recently shown to have some immunosuppressive effects in leukemia T cells [19]. Therefore the method of instance testing could be particularly valuable for the identification of pharmacological and toxicological properties in novel molecules.

## Discussion

Our results indicate that the method presented here can identify many significant connections to a query gene signature. Then what criteria should we use and which compounds should we choose if new biological hypotheses are to be developed? Our suggestion is to concentrate more on those compounds which have many replicate instances in the database. Because the results obtained for those big treatment sets do not depend heavily on the quality of a small number of treatment instances, as in the case of small treatment sets or singleton sets (treatment sets with only 1 instance each). Lamb et al also recognized the importance of having replicate instances, and noted that the power to detect connections might be greater for compounds with many replicates. In defining a treatment set, ideally only the treatment instances of the same compound with the same dose and the same cell type should be considered as a set. For example, the biological state of HL60 cells perturbed by raloxifene should be considered as a different biological state from that of MCF7 cells perturbed with the same compound, thus these two instances of raloxifene should not be put into the same set. In this paper for comparative purposes we adopted Lamb et al's choice in defining a treatment set, i.e., all the instances of a compound were grouped together as a set regardless of their possible differences in dose or cell type. Mixing the instances of a compound with different doses and/or different cell types increases the heterogeneity of an otherwise more homogenous treatment set. This tends to average out the distinct characteristics attributable to the cell type or dosage difference, making some set-level connections insignificant or their interpretation difficult. In such cases the instance level connections supported by statistical significance can be of great help in interpreting the results. For future connectivity maps, efforts should be made to provide as many replicate treatment instances (replicates with the same compound, the same dose, and the same cell type, etc) as possible, so that the undesirable reliance on individual instances can be minimized.

The successful identification of real biological connections between the reference profiles and a gene signature also depends on the quality of the gene signature. In this paper, all the three experimentally derived signatures were compiled from independent studies, in which the original authors had selected those genes as most relevant to characterize the biological states being studied. It is reasonable to ask though how many genes should be selected from the full list of genes on the microarray to best characterize a biological state? We believe this will depend on the specific biological condition being investigated, and is the decision of the individual investigator. It is expected that only those genes that show significant differential expression should be included in the gene signature. In our experience, as also shown by the examples here, gene signatures with length in the order of 10 to 100 work well, but this can only serve as a rough guide. A Java application implementing the method presented in this paper can be downloaded via FTP from our institution's website [see Additional file 3].

## Conclusion

We have presented in this paper a framework for a new connectivity map, with the advantages that statistical significance measures are calculated at both treatment instance level and treatment set level, thus providing effective control over false connections. This important safeguard was not available in the original Connectivity Map at the instance level, as revealed by the two examples of random gene signatures. As the connectivity maps are most useful for high throughput screening and for generating new biological hypothesis, it is crucial that false connections are tightly controlled. We compared the performance of the method here with the original Connectivity Map using two gene signatures (for HDAC inhibitors and estrogens respectively) previously compiled in [1] and also a new gene signature for immunosuppressive drugs. All these examples demonstrated that our method is more sensitive and robust than the original. With its increased sensitivity and with false connections being properly controlled, the method presented here can potentially benefit biomedical researchers by saving them time and resources and increasing their rate of success in pursuing new biological hypothesis based on the findings of connectivity maps.

## Authors' contributions

SDZ and TWG designed the study. SDZ developed the algorithm, implemented the method, and analyzed the data. SDZ and TWG wrote the paper. All authors read and approved the final manuscript.

## Supplementary Material

Additional file 1 **SupplementaryData**. The full tabulated results for the 5 gene signatures discussed in the paper, which can be opened with MS Excel. Click here for file

Additional file 2 **SupplementaryInfo**. A more detailed discussion on the two different null hypotheses used in the set-level analysis. Click here for file

Additional file 3 **DownloadingSoftware-info**. Information for downloading the java application implementing the method presented in this paper. Click here for file
