# Supplementary material for: A simple and robust method for connecting small-molecule drugs using gene-expression signatures
Source: BMC Bioinformatics. 2008 Jun 2;9:258. doi: 10.1186/1471-2105-9-258 (PMC2464610; doi:10.1186/1471-2105-9-258)
Supplement: Additional file 2 — SupplementaryInfo. A more detailed discussion on the two different null hypotheses used in the set-level analysis. [file 1471-2105-9-258-S2.pdf]

Supplementary information for A simple and robust method for connecting small-molecule drugs using gene-expression signatures

Shu-Dong Zhang*[1] and Timothy W. Gant*[1]

[1]MRC Toxicology Unit, Hodgkin Building, Lancaster Road, University of Leicester, Leicester, UK

Email: Shu-Dong Zhang*- sdz1@le.ac.uk; Timothy W. Gant*- twg1@le.ac.uk;

*Corresponding author

Two different null hypotheses in the set-level analysis

In the main text we have demonstrated with the two examples of random gene signatures that on the individual treatment instance level, the original Connectivity Map does not provide effective safeguards against possible false connections. The reason for this was in the way the connectivity scores were calculated. Briefly, Lamb et al's connectivity score was based on the Kolmogorov-Smirnov statistic (see the Supporting Online Material for [1]). At first two K-S statistics were calculated, one for the up-regulated genes in the signature, and one for the down-regulated genes, then these two values were combined to give a single connectivity score. This way of defining the connection score was rather complex, which made the calculation of a possible p-value on an individual instance level difficult, if not impossible.

On the treatment set level, the Connectivity Map provides a permutation p value when a set of treatment instances associated with the same compound were viewed as a whole. The permutation p value was calculated by comparing a statistic (again based on K-S statistic, see the Supporting Online Material for [1]) of the treatment set with the distribution of many random-set statistics. Those random sets were formed by randomly selecting treatment instances from all instances. From the way the permutation p value was calculated, it was clear that the following null hypothesis was being tested: *The set of instances in question have the same pattern (distribution) of connections with the query gene signature compared with a randomly formed set of the same size.* However, this null hypothesis is not the most appropriate one to test, because it is not directly relevant to the question of whether the query gene signature had any real biological connection to the treatment set. Similar issues in a different context have been addressed in

recent studies on the significance analysis of gene sets [2–4]. Tian et al were among the first groups of authors who made explicit distinctions between two null hypotheses [2] concerning a set of entities (a set of genes in context of Tian et al's paper). In the present context, the two null hypotheses are:

1. Hypothesis Q_1: The treatment instances in a set show the same pattern of connections with the query gene signature compared with the rest of the treatment instances.

2. Hypothesis Q_2: The treatment set does not contain any treatment instances which have real connections with the query gene signature.

Tian et al's discussions over the relationship between Q_1 and Q_2 [2] apply to a treatment set as follows: Given all the 453 treatment instances, even if none of them have real connection to the query gene signature, the observed connection scores of a treatment set could still be very different from those treatment instances outside of the set because of the special correlation structure among the treatment instances within the set. Chen et al also raised some concerns with testing the hypothesis Q_1 [4], that this hypothesis does not test whether the treatment set has above-random connections to the query gene signature, but rather it tests whether the observed connections in the set are more or less than a randomly formed set of same size. We agree with Chen et al's assessment on the inappropriateness of Q_1. It was clear that the Connectivity Map was testing the null hypothesis Q_1. In our paper, we tested Q_2, which is more appropriate.

References

1. Lamb J, Crawford ED, Peck D, Modell JW, Blat IC, Wrobel MJ, Lerner J, Brunet JP, Subramanian A, Ross KN, Reich M, Hieronymus H, Wei G, Armstrong SA, Haggarty SJ, Clemons PA, Wei R, Carr SA, Lander ES, Golub TR: **The Connectivity Map: Using Gene-Expression Signatures to Connect Small Molecules, Genes, and Disease**. *Science* 2006, **313**(5795):1929–1935.
2. Tian L, Greenberg SA, Kong SW, Altschuler J, Kohane IS, Park PJ: **Discovering statistically significant pathways in expression profiling studies**. *PNAS* 2005, **102**(38):13544–13549.
3. Efron B, Tibshirani R: **On testing the significance of sets of genes**. *Ann. Appl. Statist.* 2007, **1**:107–129.
4. Chen JJ, Lee T, Delongchamp RR, Chen T, Tsai CA: **Significance analysis of groups of genes in expression profiling studies**. *Bioinformatics* 2007, **23**(16):2104–2112.
